# Design and Performance of a Metal-Shielded Piezoelectric Sensor

**DOI:** 10.3390/s17061284

**Published:** 2017-06-04

**Authors:** Álvaro Sáenz de Inestrillas, Francisco Camarena, Manuel Bou Cabo, Julián M. Barreiro, Antonio Reig

**Affiliations:** 1 Institut d’Investigació per a la Gestió Integrada de les Zones Costaneres (IGIC), Escola Politècnica Superior de Gandia (EPSG), Universitat Politècnica de València (UPV), 46730 Grau de Gandia, Spain; alsaedei@epsg.upv.es (A.S.d.I.); manuelbcabo@gmail.com (M.B.C.); jbarreiro@academia.usbbog.edu.co (J.M.B.); 2 Instituto de Instrumentación para Imagen Molecular (i3M), Universitat Politècnica de València (UPV), Consejo Superior de Investigaciones Científicas (CSIC), 46022 València, Spain; 3 Departamento de Física Aplicada, Universitat Politècnica de València (UPV), 46022 València, Spain; areig@fis.upv.es

**Keywords:** acoustic sensor, piezoelectric ceramic, encapsulated transducer, piezoelectric FEM simulation, sensitivity, explosive atmospheres

## Abstract

In certain circumstances when acoustic measurements are required in the presence of explosive atmospheres the sensor must be placed inside a Faraday Cage. Piezoelectric active materials are suitable for this purpose as they do not need an electrical power supply, although the metal shielding can considerably reduce sensor sensitivity, which is already low at the acoustic frequency range (<20 kHz). This paper describes a metal-shielded piezoelectric sensor designed to work in the range of frequencies between 1 and 2 kHz and in these environmental conditions. The main idea was to add a thin material layer to the front face of the piezoelectric ceramic in order to force the system to vibrate in flexure mode at low frequencies. The resonant frequency and sensitivity of the system was studied as a function of the radius, thickness, and material of the thin layer. The study includes a comparison of theoretical model, FEM simulation, and real data measured using three aluminum and three steel prototypes of different sizes.

## 1. Introduction

In industry, fluids are often stored in different types of tanks. With the passage of time these tanks may suffer small cracks through which the fluids can leak. These leaks can be costly, but can be detected by pressure differential techniques and acoustically detecting the sound created due to the passage of air through any perforation that may be present, or the bubbles that are formed in the liquid stored in the container as a result of the passage of air [1,2]. In a case such as a biofuel tank with an atmosphere highly unstable to electromagnetic waves, the sensor must be placed inside a Faraday Cage. Depending on the application considered, the frequency of interest can range from hundreds of Hz to kHz, and the amplitude of the signals may vary by several orders of magnitude [3,4,5]. Therefore, having a good signal-to-noise ratio capable of recording low intensity signals from system malfunctions is more important than a linear response from the sensor.

Several types of sensors can function in the frequency range desired, including electrostatic microphones [6,7], which offer high quality in terms of sensitivity, but have a serious restriction as they require an extremely dangerous bias voltage under explosive conditions. Magnetic induction based sensors require the relative movement between the coil and the magnet caused by the acoustic wave to be detected, but this is difficult to achieve in a metal-shielded sensor, so that the signal-to-noise ratio can be very low [8]. Micro Electro-Mechanical Systems (MEMS) are commonly used for monitoring purposes in industry [9], and Piezoelectric Micromachined Ultrasound Transducer (PMUT) technology can achieve high sensitivity values [10], but transduction techniques rely on cells with thin-stretched membranes, which reduces robustness and makes them unsuitable for some industrial applications, as it is our case. Other types of technologies, such as electret condenser microphones, could be used considering frequency response and directivity, but they usually have worse performance than those mentioned above, even in non-aggressive environments [11].

In this context, piezoelectric sensors can be considered as a possible solution to deal with the problem of capturing low frequency acoustic signals with enough sensitivity to maintain a proper signal-to-noise ratio in explosive environments. Piezoelectric transducers offer numerous advantages such as high transduction efficiency, excellent ruggedness, fast response time, free electromagnetic interference, and low cost [12]. To increase sensitivity at low frequencies, a unimorph design should be considered in which flexural modes force the piezoelectric ceramic to vibrate outside its natural resonance frequency [13]. The simplest design is formed by a single active element, which can be the piezoelectric ceramic attached to a metal diaphragm (buzzer). The geometric shape of the plate and the thickness and stiffness of the material are important elements in improving the sensitivity and ruggedness of the output signal [14].

This paper proposes several unimorph piezoelectric sensors able to operate in explosive media thanks to their metal housing, with the main objective of obtaining good sensitivity between 1 and 2 kHz, which is the signal bandwidth detected for a 0.2 mm diameter leak after applying a −200 mbar decompression in our tank. The bandwidths of the sensors are not optimized, as they are designed to work as trigger systems. The design process involves a physical model for the flexure of the plate and Finite Element Method (FEM) simulations to evaluate the effect of the piezoelectric ceramic attached to the plate in the resonance frequency and sensitivity. To validate the designs, the experimental measurements of six prototypes of the sensors are given.

The paper is structured as follows: in Section 1 we outline the physical problem to be solved and the available alternatives. Section 2 describes the physical model of the vibration plates on which this work is based. Section 3 defines the sensor design, materials, and methods to characterize the sensors both numerically and experimentally. Section 4 offers the results of the six prototypes built and Section 5 contains our conclusions.

## 2. Physical Model

Unimorph piezoelectric sensors that respond to signals in the audible range (1 to 10 kHz) vibrate in a flexural mode [15]. As our sensors must be metal-shielded, advantage can be taken of the natural vibration of part of the metal structure itself, in particular the front circular plate (front-plate) and the adhered piezoelectric ceramics can be made to vibrate close to its flexural mode. The eigenfrequencies of a circular plate clamped to its ends can be calculated analytically. These frequencies depend on the stiffness of the plate itself, as well as on the volume density of the material and its dimensions, and are given by the expression [16]

(1) $$f_{n}=\frac{g_{n}^{2}}{2\pi }\frac{d}{\sqrt{12}}{\left(\frac{\gamma }{\rho \left(1-\sigma^{2}\right)}\right)}^{\frac{1}{2}}$$

where $f_{n}$ is the frequency corresponding to mode n, d is the thickness of the plate, γ is the Young’s modulus of the material, ρ is the volume density of the material, σ is the Poisson coefficient, and $g_{n}\approx \frac{n\pi }{a}$ where a is the plate radius.

Considering an aluminum metal plate with d=0.3 mm and r=10.0 mm, the first resonance mode is $f_{1}=7598\;\mathrm{Hz}$. For robustness considerations the thickness of the metal plate of our sensors will be fixed to 0.3 mm, so a little bit higher radius could be interesting to reduce the resonant frequency and improve the sensitivity in the range of interest (1–2 kHz). Therefore, although the ceramics used are not designed to work within that range in their thickness mode, the movement of the plate interacting with the acoustic wave causes a deformation in the piezoelectric ceramic, thereby obtaining a voltage difference.

In the following sections, this hypothesis is tested through simulations and various designs are made.

## 3. Design and Methods

### 3.1. Design

**Figure 1:**
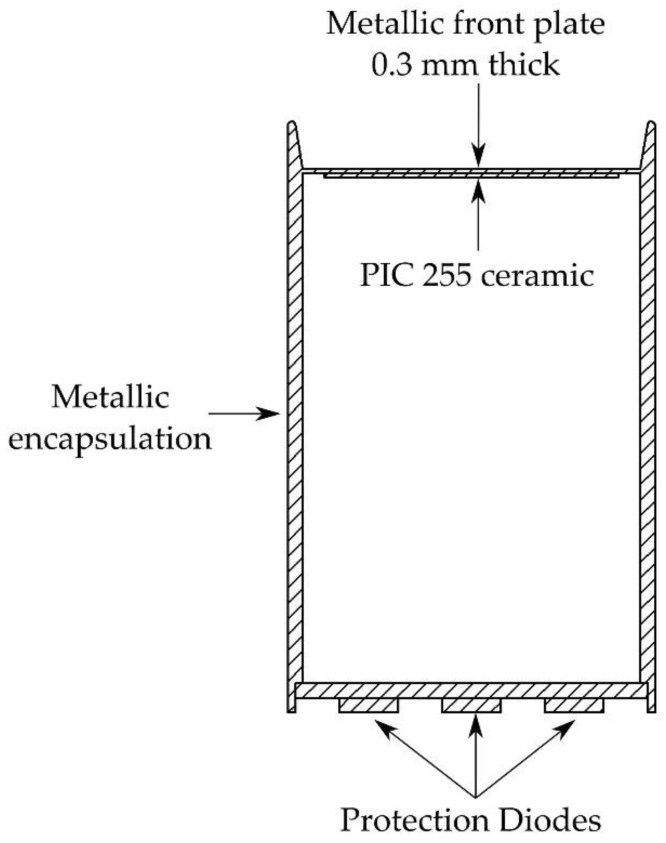
Design scheme of the sensor.

The sensor design is based on a cylindrical aluminum (density 2700 kg/m^3^, Young’s module 70 GPa and Poisson’s ratio 0.33) or stainless steel (density 7850 kg/m^3^, Young’s modulus 200 GPa and Poisson’s ratio 0.33) Faraday Cage (lateral thickness 1.0 mm, height 40.0 mm, variable radius from 10.0 to 19.0 mm). At one end of the cylinder there is a circular plate (variable thickness from 0.1 to 0.5 mm, variable radius from 10.0 to 19.0 mm), which receives acoustic waves and makes the piezoelectric element vibrate. PIC 255 Modified Lead Zirconate-Lead Titanate piezoelectric ceramics (0.3 mm thickness and 10 mm radius) from PI Ceramic (Lindenstrasse, Lederhose, Germany) were used. These piezoelectric ceramics are considered soft PZT materials (ML-Standard DOD-STD-1376A type-II) with a low quality factor (~80) which make them suitable for microphones [17]. A conductive epoxy (CircuitWorks CW2400, Chemtronics, Kannesaw, GA, USA) was used to glue the piezoelectric ceramic and the front-plate, so that the ground is common to the structure that serves as shielding and the negative pole of the piezoelectric ceramic. To protect against high-level electromagnetic signals from the ceramic, especially signals produced by accidental knocks on the sensor, SMCJ36(C)A transient voltage suppressor diodes from Fairchild Semiconductor (Sunnyvale, CA, USA) were used at the output of the piezoelectric element [18]. Figure 1 shows the sensor cross section.

### 3.2. Experimental Set-Up

**Figure 2:**
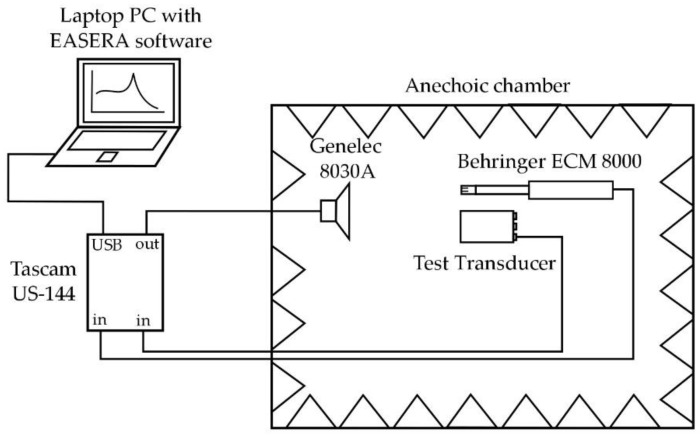
Experimental set up for sensitivity measurements.

The electrical impedance of the piezoelectric ceramic was measured with an impedance analyzer (Wayne & Kerr 6500p, Wayne & Kerr Electronics, Bognor Regis, West Sussex, UK) as described in UNE-EN61094-8-2013 [19]. This standard uses a sine-sweep signal and a windowed impulse response to compare the sensor under study with a previously calibrated sensor (Behringer ECM 8000, Behringer, Willich, Germany), which has a flat frequency response at the frequencies of interest [20]. A self-powered loudspeaker (Genelec 8030A, Genelec, Lisalmi, Finland) with a flat frequency response [21] was used as an emitter. A 96 kHz sampling frequency Tascam US-144 (Montebello, CA, USA) card [22] was used for data acquisition. All the devices were controlled by means of a laptop PC with EASERA software (AFMG Technologies, Berlin, Germany) [23] to calibrate the acquisition card and the reference microphone. The measurement process was carried out in an anechoic chamber (cutoff frequency 70 Hz) to avoid sound reflections. Figure 2 shows a scheme of the equipment configuration during the measurements.

### 3.3. Finite Element Simulation

**Figure 3:**
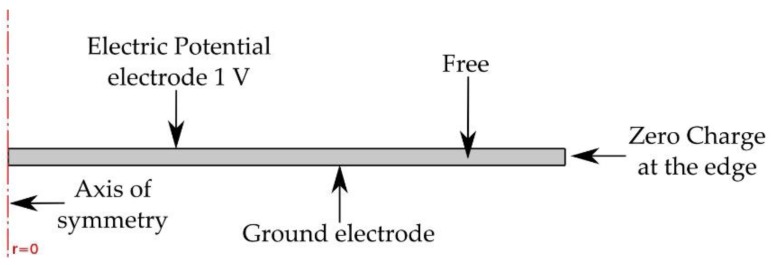
Model for the simulation of the piezoelectric element’s electrical response.

COMSOL Multiphysics 4.4 software package (COMSOL, Stockholm, Sweden) [24] was used to simulate the mechanical resonance, electrical impedance, and sensitivity of the system shown in Figure 1. Although Equation (1) provides the values for the set of flexural modes of the sensor’s front plate, the numerical simulations provide a more realistic approach, as they incorporate the effect of the glued piezoelectric element (inertial mass), as well as other possible resonant modes associated with the sensor’s mechanical structure. The COMSOL piezoelectric module [24] can simulate the electrical response of a system with piezoelectric elements by solving the piezoelectric constitutive equations [25]. Figure 3 shows the piezoelectric ceramic model used to obtain the vibration modes of a free disc, where 1 V is applied to the positive pole as a boundary condition, according to the experimental procedure of our impedance analyzer. The zero charge at the edge is required for free movement. The electrical current flowing through the piezoelectric material is obtained and thus also the electrical impedance at the terminals of the piezoelectric element [26,27].

**Figure 4:**
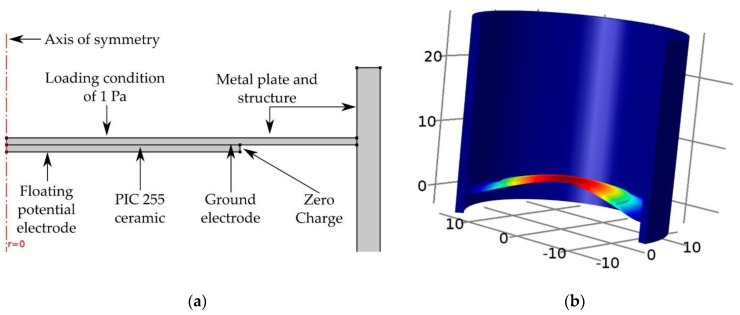
(**a**) Model for the simulation of sensor sensitivity; (**b**) Simulated first flexural mode of the front plate with the piezoelectric ceramic attached.

To obtain the sensitivity of the whole sensor, a pressure of 1 Pa at the frequencies of interest (1 kHz to 10 kHz) is exerted on the metal front-plate as a boundary condition. This pressure changes the metal and piezoelectric ceramic shapes, generating a voltage at the terminals of the piezoelectric ceramic. This voltage is placed as a floating potential or terminal condition. Figure 4a shows the schematic configuration of the simulation, and Figure 4b shows the simulated first flexural mode of the front plate with the piezoelectric ceramic attached.

## 4. Results and Discussion

### 4.1. Mechanical Resonance Frequencies of the System

**Figure 5:**
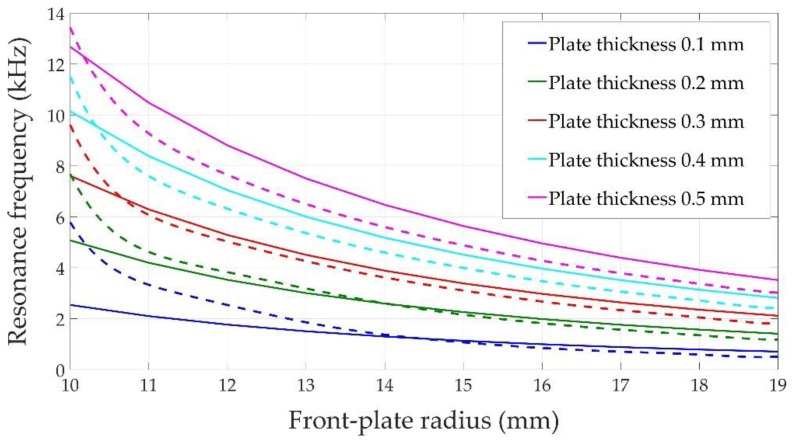
Flexural first mode resonance frequency of a circular aluminum plate obtained analytically (solid lines) and numerically with a piezoelectric ceramic attached (dashed lines).

Figure 5 shows the flexural resonant frequency of the sensor shown in Figure 1, obtained both analytically (front-plate only) and numerically (front plate plus piezoelectric ceramic) for different design configurations. Aluminum was considered as the structural material. The thickness of the front-plate varies between 0.1 mm and 0.5 mm and its radius between 10.0 and 19.0 mm. The ceramic dimensions are fixed at a radius of 10.0 mm and thickness of 0.3 mm.

According to Equation (1), the first flexural mode resonance frequency increases with front-plate thickness and decreases with a larger front-plate radius (solid lines in Figure 5). A similar behavior can be observed in the simulated results (dashed lines in Figure 5), although the analytical and numerical values do not match exactly due to the effect of the attached piezoelectric element. When the radius of the front-plate matches the radius of the piezoelectric ceramic, i.e., 10.0 mm, the system acts as if it had a thicker front-plate and the resonance frequency increases considerably (as predicted by Equation (1)). The piezoelectric element increases the stiffness of the system. This effect is not so important when the radius of the front-plate is bigger than the radius of the piezoelectric ceramic, and once both curves intersect, the piezoelectric ceramic acts more as an attached inertial mass and less as a constitutive element. Therefore, the natural resonance frequency of the system is lower than the one for the clamped ends plate [16]. The point where both curves intersect occurs later for lower front-plate thicknesses, because in these cases the piezoelectric ceramic provides the main stiffness to the system. Our main conclusion is that the resonant frequencies of the designed sensors fall into the audible range and, therefore, metal-shielded piezoelectric sensors can be effective at these frequencies.

### 4.2. Electric Response of the Piezoelectric Ceramics

**Figure 6:**
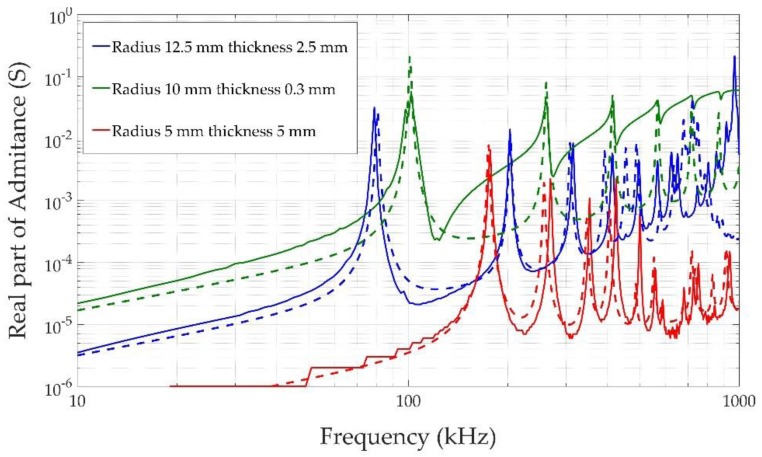
Experimental (continuous line) and simulated (dashed line) admittance for three freely vibrating piezoelectric ceramics.

Figure 6 shows the numerical (dashed curves) and experimental (solid curves) results obtained for the electrical response of three vibrating piezoelectric ceramics with different radiuses and thicknesses with free borders. As can be seen, the different thicknesses and radial modes resonate at frequencies between 100 and 1000 kHz, which are far removed from the acoustical frequency range of interest for our sensors. The simulation based on COMSOL with the piezoelectric module is able to reproduce most of the resonant modes, although the admittance value outside the resonance is overestimated in the simulation, which could have been be due to the difficulty in adjusting all the piezoelectric ceramic parameters in the simulation.

### 4.3. Sensitivity Response of the System

Six physical prototypes were built according to the conclusions of the simulations and the robustness requirements for the device, i.e., three aluminum and three stainless steel devices with 0.3 mm thick front-plate and radius: 11.5, 15, and 17.5 mm.

**Figure 7:**
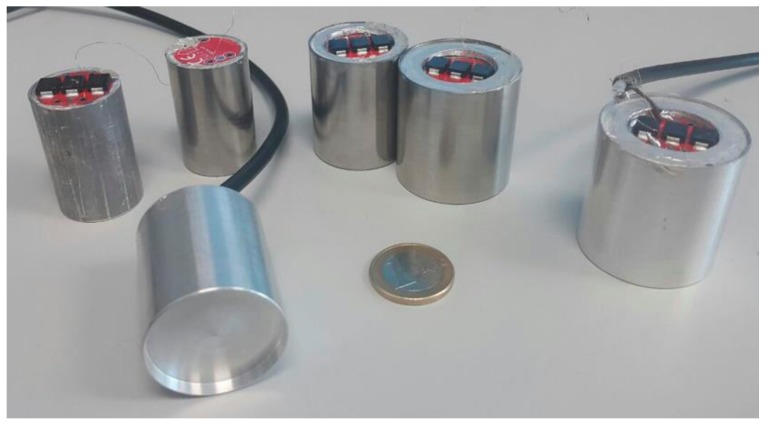
Three aluminum and three stainless steel sensors with 0.3 mm front-plate thickness and radius: 11.5, 15, and 17.5 mm. Each device is supported by a cylindrical structure 1.0 mm thick and 40.0 mm long. The ceramic material used in each device is PI Ceramics 255 with a 10.0 mm radius and 0.3 mm thick.

**Figure 8:**
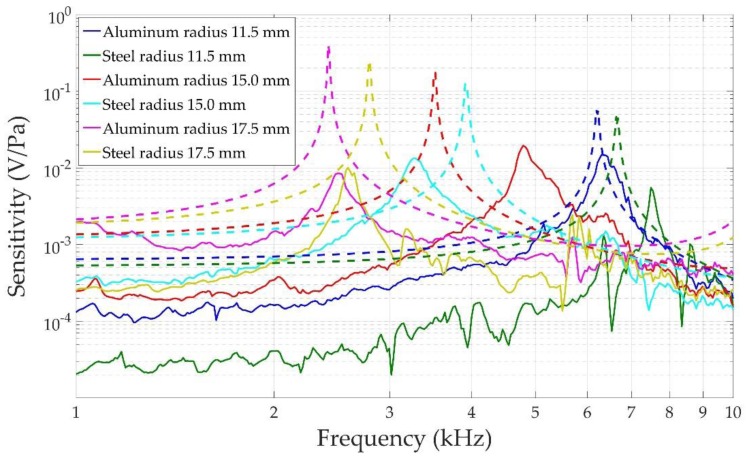
Measured sensitivity (solid lines) and simulated sensitivity (dashed lines) of the six sensors.

The assembled final sensors can be seen in Figure 7. Their measured and simulated sensitivity results are shown in Figure 8. Both experiments and simulations show the same trends, considering possible defects in their construction and in the bonding of the piezoelectric ceramic to the front-plate. It should also be remembered that the conductive epoxy used to glue the piezoelectric ceramic to the front-plate was not simulated. Several conclusions can be drawn: (a) the sensors have a very narrow bandwidth at their resonance peak (always between 2 and 10 kHz), which is due to the high quality of the vibrating system: front-plate plus piezoelectric ceramic; (b) as Equation (1) predicted, the resonance frequency decreases as the radius of the front-plate increases; (c) for the same radius, aluminum resonates at lower frequencies than steel because of its lower Young’s modulus; (d) simulation and experimental results show the same behavior and match well, taking into account that the six devices were assembled manually and affect the final operation of the sensor, such as the bonding of the ceramic to the front-plate or the final quality of the metal shield. Yellow and green curves in Figure 8 (corresponding to the 17.5 mm radius steel sensor and the 11 mm radius steel sensor) are especially noisy, indicating a poor sensor finish. This implies a lower sensitivity and makes difficult to extract any conclusion from them. The automated assembly of the sensors would help to homogenize their final behavior.

Typical microphones work in the frequency range far below their resonance frequency, where they have a flat response [10]. This is not the case of our sensors in the region between 1 and 2 kHz, where sensitivity changes from 2 to 8 mV/Pa (numerical result for aluminum front-plate with a radius of 17.5 mm). Nevertheless, our goal was not to obtain a flat response, but to get the best possible sensitivity between 1 and 2 kHz. In order to get this optimum sensitivity, the designed sensors should resonate with a flexural mode in the front-plate just above the region of interest, i.e., between 2 and 3 kHz in our case. For this, the best choice would be the sensors with a 17.5 mm radius (preferably of aluminum), as can be seen in Table 1, which shows the mean sensitivity between 1 and 2 kHz for the different configurations. Table 1 shows that sensitivity increases with the front-plate radius. The differences between numerical and experimental values are due to the lack of reproducibility of the manufacturing process of the sensors.

Sensitivity of the sensors depends on the capacity of the acoustic wave to deform the piezoelectric element. To increase this deformation, the sensors have been tuned to resonate in a flexural mode with a frequency above the frequency range of interest by adjusting their front plate radius. The material of the front plate also plays a role in sensitivity. First, it affects the value of the resonance frequency through its Young’s Modulus, as Equation 1 predicts. If we look at the simulated curves in Figure 8, we see that the aluminum resonant peak is always below the steel resonant peak for a fixed radius. It implies a higher sensitivity in the 1–2 kHz frequency range, as the resonance is closer in each case. Second, a lower Young’s Modulus implies higher displacements when material is subjected to an external force, so that higher displacements are expected in the aluminum front plate than in the steel one when the same acoustic wave reaches the sensor. This effect can also be observed in Figure 8, particularly in the simulated results, which do not suffer the uncertainty of the building procedure of the sensors. For a fixed radius, the aluminum peak sensitivity is always slightly higher than the steel peak sensitivity.

## 5. Conclusions

We evaluated the acoustical sensitivity of a metal-shielded piezoelectric transducer designed to work as an acoustical trigger sensor in explosive atmospheres in the frequency range between 1 and 2 kHz. The function of the metal shield is to act as a Faraday Cage and protect the explosive atmosphere from electrical fields. As the high acoustical impedance of the metal considerably reduces the acoustical energy that can reach the piezoelectric ceramic, a new design based on the use of a flexural mode of a part of the Faraday Cage structure is proposed (circular front-plate) in order to improve the sensitivity of the sensor. The piezoelectric ceramic is attached to the front-plate, forcing both to vibrate together in a resonance frequency close to the region of interest. It has been shown numerically that the ratio between the front-plate radius and that of the piezoelectric ceramic determines the influence of the latter on the front-plate resonance frequency. Two phases can be distinguished: the first (similar radius) in which the piezoelectric ceramic increases the stiffness of the front-plate and consequently the resonance frequency of the system, and the second (piezo radius smaller than plate radius) in which the piezoelectric ceramic acts as an inertial mass, reducing the resonance frequency of the system. A parametric study of the dimensions of the front-plate (radius and thickness) was carried out with finite elements simulation to maximize the sensitivity of the sensor between 1 and 2 kHz. Six aluminum and steel sensors were built according to the information provided by the numerical study. Their sensitivity was measured and compared with the simulation. The results show that in order to obtain the best possible sensitivity in the range of frequencies between 1 and 2 kHz, the radius and thickness of the front-plate has to be adjusted (according to the material used) to obtain a flexural mode between 3 and 4 kHz. Future studies will try to obtain a flat sensor response while keeping sensitivity high.

## Figures and Tables

**Table 1:** Mean sensitivity of the six prototypes between 1 and 2 kHz.

Transducer	Measurement (mV/Pa)	Simulation (mV/Pa)
Aluminum (radius 17.5 mm)	1.20	3.30
Aluminum (radius 15 mm)	0.23	1.60
Aluminum (radius 11.5 mm)	0.14	0.67
Steel (radius 17.5 mm)	0.33	2.50
Steel (radius 15 mm)	0.39	1.40
Steel (radius 11.5 mm)	0.03	0.55
